# Deep neural network based histological scoring of lung fibrosis and inflammation in the mouse model system

**DOI:** 10.1371/journal.pone.0202708

**Published:** 2018-08-23

**Authors:** Fabian Heinemann, Gerald Birk, Tanja Schoenberger, Birgit Stierstorfer

**Affiliations:** Target Discovery Research, Boehringer Ingelheim Pharma GmbH & Co. KG, Biberach an der Riß, Germany; University of Oklahoma Health Sciences Center, UNITED STATES

## Abstract

Preclinical studies of novel compounds rely on quantitative readouts from animal models. Frequently employed readouts from histopathological tissue scoring are time consuming, require highly specialized staff and are subject to inherent variability. Recent advances in deep convolutional neural networks (CNN) now allow automating such scoring tasks. Here, we demonstrate this for the case of the Ashcroft fibrosis score and a newly developed inflammation score to characterize fibrotic and inflammatory lung diseases. Sections of lung tissue from mice exhibiting a wide range of fibrotic and inflammatory states were stained with Masson trichrome. Whole slide scans using a 20x objective were acquired and cut into smaller tiles of 512x512 pixels. The tiles were subsequently classified by specialized CNNs, either an “Ashcroft fibrosis CNN” or an “inflammation CNN”. For the Ashcroft fibrosis score the CNN was fine-tuned by using 14000 labelled tiles. For the inflammation score the CNN was trained with 3500 labelled tiles. After training, the Ashcroft fibrosis CNN achieved an accuracy of 79.5% and the inflammation CNN an accuracy of 80.0%. An error analysis revealed that misclassifications are almost exclusively with neighboring scores, which reflects the inherent ambiguity of parts of the data. The variability between two experts was found to be larger than the variability between the CNN classifications and the ground truth. The CNN generated Ashcroft score was in very good agreement with the score of a pathologist (r^2^ = 0.92). Our results demonstrate that costly and time consuming scoring tasks can be automated and standardized with deep learning. New scores such as the inflammation score can be easily developed with the approach presented here.

## Introduction

Results from animal models are a necessary step in preclinical development for the study of novel compounds. In the case of fibrotic pulmonary diseases, such as idiopathic pulmonary disease (IPF) [1], a widely used model system is the bleomycin model of lung injury in the mouse. [2], [3] In this model fibrosis is artificially induced by administration of bleomycin and any reduction of fibrotic burden by treatment is compared to a placebo treatment. Inflammatory diseases are also studied using artificially induced inflammation of the lung. Common models are based on the administration of inflammatory agents such as lipopolysaccharide (LPS)[4], or the exposure to cigarette smoke, [5], [6] with the goal of decreasing the amount of inflammation in the lungs.

To evaluate the efficacy of a compound in preclinical testing in a statistical manner it is crucial to quantify the changes in the lung tissue. In case of fibrotic changes, the Ashcroft fibrosis score is widely used.[7] Ashcroft’s lung fibrosis score is traditionally performed by a pathologist using a 10x objective in multiple fields of view. For each field of view the Ashcroft score can be assigned to discrete values from 0–8 with 0 corresponding to a healthy lung and 8 to “total fibrous obliteration of the field”. [7] However, the latter value is typically not found in our experimental settings.

A problem with Ashcroft scoring is that the scoring procedure requires the time of highly trained pathologists and is subject to intra- and inter-observer differences. Automation of Ashcroft scoring with image analysis of lung microscopy images or micro-CT images was hampered by the lack of resolution of these methods in the therapeutically important lower range from 0–3.[8] [9] Other approaches combined computer vision and machine learning to quantify fibrotic changes in lung tissue [10], [11], [12]. Despite their usefulness, these methods resulted in self-defined values and lack comparability to, for instance, the more widely accepted Ashcroft score. In addition, sufficient resolution when fibrotic changes first occur and morphological changes are subtle was not shown.

There is no generally available consensus score available regarding inflammatory changes, which hampers the comparability of studies. The quantification of inflammatory changes by image analysis is particularly challenging due to the large variety of possible inflammatory morphologies.

Recently convolutional neural networks (CNNs), which are a form of deep learning, revolutionized several image processing tasks.[13] In the object recognition task [14], [15], [16] the best CNNs are assumed to have surpassed human performance. [17] Similarly, CNNs are rapidly advancing in object localization [18] and segmentation tasks.[19] Currently, these technologies are applied to a wide range of biomedical applications. Examples are the diagnosis of malignant melanoma based on photographs with a comparable performance to a dermatologist [20] or the identification of metastatic breast cancer from microscopy images.[21] It is evident that also in histopathology an increasing number of image analysis tasks will be done by using forms of deep learning.[22]

Here, we demonstrate for the Ashcroft fibrosis score that deep learning can be used to obtain scores with a comparable performance as a pathologist. Moreover, we developed a novel score to quantify the degree of inflammation based on the density of immune cells. In both cases non-alveolar tissue, such as lymph nodes, large vessels, fat or large bronchi are automatically recognized by the deep neural network and excluded from further analysis. In addition, our deep learning based scores can be used to generate spatially resolved maps of fibrosis (Ashcroft score) and inflammation.

## Materials and methods

### Animals

For this work, lung sections of previous animal studies run between 2015 and 2017 were used and reanalyzed. To minimize a possible bias, mice of different sex, age, vendors and also transgenic mice were used. C57BL/6J mice, C57BL/6N mice, and transgenic mice on C57BL/6N background were purchased from Charles River (Sulzfeld, Germany), Taconic (Denmark) or Janvier (France). The studies included young (8 to 12 week old) and old (8–10 months and 18–20 months) mice. Animals were maintained in accordance with German national guidelines, legal regulations and the guidelines of the Association for Accreditation of Laboratory Animal Care and experiments performed after permission by the Regierungspräsidium Tübingen, Germany.

### Induction of fibrosis with bleomycin

Animals were anaesthetized using isoflurane (3–5%), 0.5–1 mg/kg bodyweight (bw). Bleomycin (Calbiochem, Darmstadt, Germany) was dissolved in sterile isotonic saline. This solution (2ml/kg bw) was intratracheally instilled at the start of the study. Control animals received a saline solution.

### Induction of inflammation in a cigarette smoke model

Mice were exposed to the cigarette smoke (CS) of five cigarettes daily inside a perspex box for 3 weeks and 12 weeks, respectively, as described in detail previously. [6] Control animals were exposed to room air.

### Lung tissue samples and staining

Animals were sacrificed by an overdose of pentobarbital i.p. and lungs were excised. After cannulation of the trachea the lungs were inflated with 4% paraformaldehyde (PFA) for 20 min at a pressure of 20 cm H_2_O. The filled lungs were then sealed by a ligature and immersed in 4% PFA for at least 24 h. Subsequently, fixed lungs were embedded in paraffin according to established protocols. 3μm sections of lung tissue were stained with Masson´s trichrome.

### Manual Ashcroft scoring procedure

Fibrosis scoring was performed by a blinded pathologist according to the established protocol by Ashcroft.[7], [23] Briefly, the lungs were analyzed with a brightfield microscope (Carl Zeiss, Jena, Germany) in 10x magnification. For each field of view the pathologist assigned the Ashcroft score. At the end the mean Ashcroft score was calculated from all fields of view.

### Microscopy for digital imaging

A Zeiss AxioScan Z1 (Carl Zeiss, Jena, Germany) whole slide scanner was used in bright field illumination using a 20x objective. Whole slide images were converted to tiff with 50% downscaling and sliced into tiles of 512x512 pixels with a resolution of 0.44 μm/pixel using Halcon image processing software (MVTec Software GmbH, Munich, Germany). This tile size was an empirical trade-off between the need for sufficient tissue visible for labelling of a tile by the pathologist (i.e. larger tiles) and the desire for a high spatial resolution (i.e. smaller tiles).

### Deep learning

Deep learning scripts were developed in Python 3.6 using the deep learning library Keras [24] based on Google’s Tensorflow library. The Keras implementation of Google’s InceptionV3 CNN architecture was used. [16] InceptionV3 was used as classifier architecture, since it has a very good classification performance on ImageNet, while being computationally efficient [25] Presumably, other modern CNN architectures such as ResNet[26] may lead to comparable results. The network was pre-trained on approximately 1.2 million images from ImageNet [27] and re-trained on the microscopy images relevant for the task described here. The final 1000 class layer for ImageNet was removed and replaced by a softmax output with 6 (Ashcroft fibrosis model) or 5 (inflammation model) classes respectively. This process of pre-training on a generic large dataset (containing images of everyday objects) and retraining on a much smaller dataset with replacing the classification layer is referred to as “transfer learning”. Briefly, the pre-training on the large ImageNet dataset ensures the emergence of convolutional filters (e.g. edge, gradient and pattern detectors). These filters are mostly generic for computer vision tasks (especially in the early CNN layers) and can be used to train a pre-trained CNN in a second step with much fewer data compared to training from scratch.

Training tiles were automatically resized by Keras from 512x512 to 299x299 pixels (the fixed input dimension of the Inception-V3 CNN). Image augmentation was applied to enhance the variety of the training data. Each image was randomly rotated by -45° to 45° and vertically flipped. Training on all layers was performed using a stochastic gradient descent optimizer using an initial learning rate lr = 0.5 10^−4^, a momentum of 0.9 and a learning rate decay of a factor 1/5 down to lr = 10^−7^ if the loss on the validation data did not reduce for three consecutive epochs. Class imbalances in the training data were compensated by oversampling.

**Fig 1:**
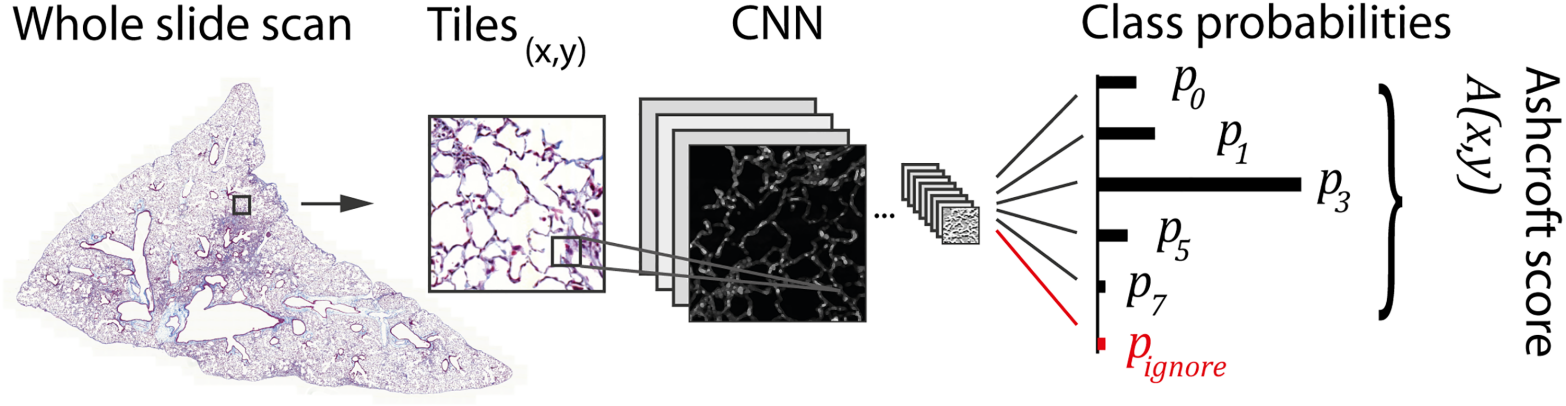
Workflow to obtain histological scores from microscopy images of murine lungs by using convolutional neural networks (CNN). We built two types of models: a CNN to classify the Ashcroft score (used as an example in the figure) and a CNN to classify an inflammation score. A whole slide scan of a mouse lung (left) is divided into smaller image tiles. The tiles are fed into a CNN model and a probability distribution over the image classes is obtained as an output. We used the Inception-V3 CNN architecture, pre-trained on the Image-Net dataset (1.28 10^6^ images) and re-trained on labelled tiles of lung tissue (between 3.5 10^3^ and 1.4 10^4^ images, see Methods). From the probability outputs of the two neural networks, the Ashcroft fibrosis and inflammation scores are computed as the score-weighted sum of the class probabilities after a renormalization to 1 without *p*_*ignore*_ (see Methods).

The output of the CNN is a probability vector *p* per tile normalized to 1 (see Fig 1). For the Ashcroft fibrosis CNN:

$${p=(p_{0},p_{1},\;p_{3},\;p_{5},\;p_{7},p_{ignore})}^{T}$$

To compute the value of the Ashcroft score from this, we only considered fields were the *p*_*ignore*_ probability was not the largest component of the vector, otherwise the tile was ignored. Subsequently, we converted the remaining probabilities to renormalized values ${\tilde{p}}_{i}$ with a sum of 1 without *p*_*ignore*_:

$${\tilde{p}}_{i}=p_{i}/(p_{0}+p_{1}+p_{3}+p_{5}+p_{7})$$

Finally, the Ashcroft score was computed as the weighted sum of the renormalized probabilities:

$$A=\sum_{i}{i\tilde{p}}_{i}$$

For the inflammation score the same procedure with classes 0, 1, 2, 3, and ignore was used. The training and classification was performed on an NVidia GTX 1080 graphics card (NVidia, Santa Clara, CA, USA). Typical training duration on an NVidia GTX 1080 was 2h 20min (~14000 tiles, Ashcroft fibrosis model, 45 epochs). In comparison, training on an Intel Xeon E5-2630, 2.2 GHz CPU took 41 hours (Intel, Santa Clara, CA, USA). Using multiple GPUs did not considerably decrease training times, since image augmentation and data transfer to the GPU became bottleneck processes in this case and the GPUs could not be operated at full capacity.

Data visualization with t-distributed Stochastic Neighbor Embedding (t-SNE) [28] was performed using the last hidden layer of the CNN and the Scikit-learn [29] implementation of t-SNE in Python.

Deep learning scripts for training and prediction are available on an Open Science Framework repository under https://osf.io/28qbc/ (DOI 10.17605/OSF.IO/28QBC).

### Data labelling

**Fig 2:**
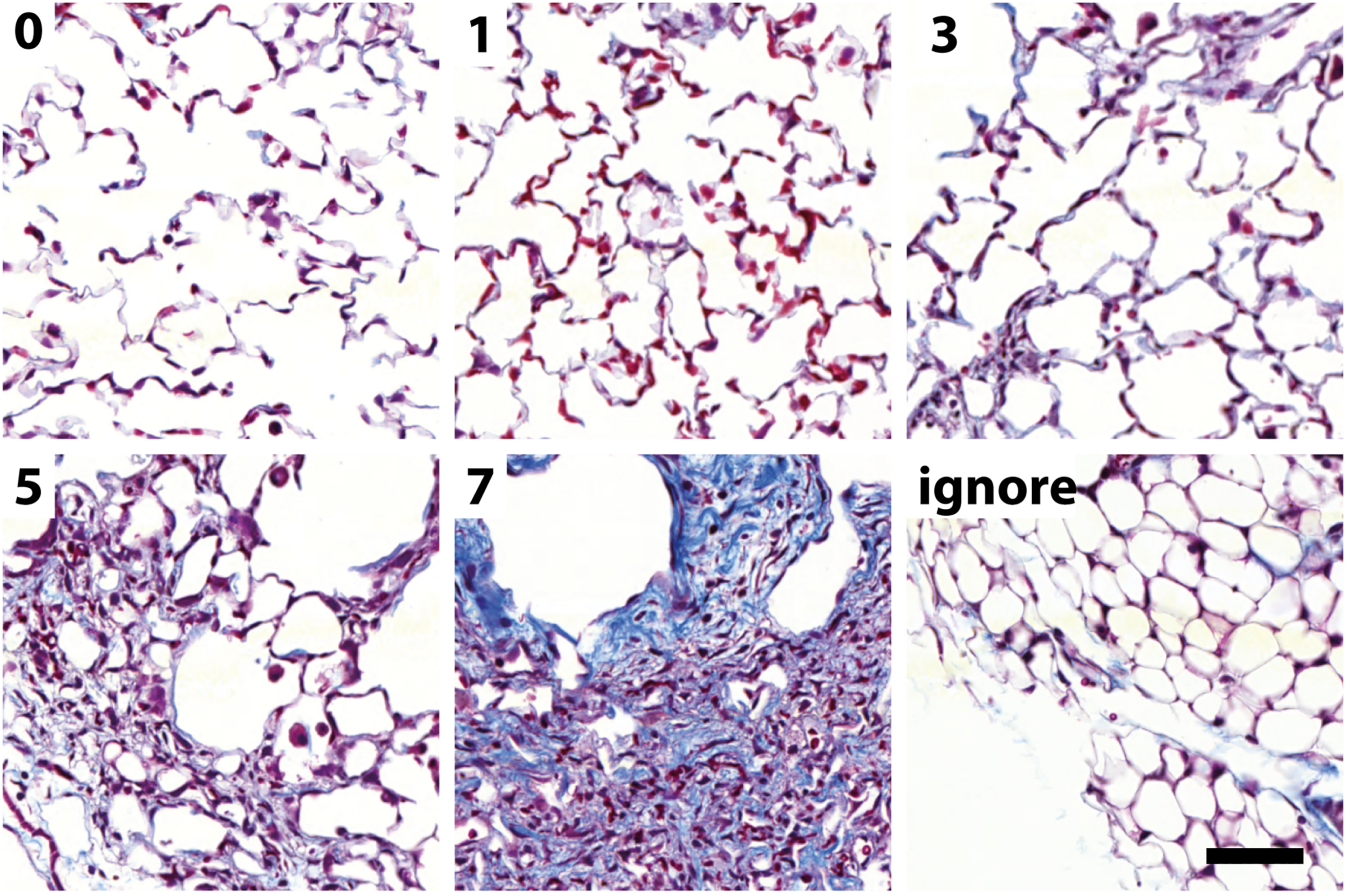
Example tiles used for the Ashcroft fibrosis score CNN. The value on the top left indicates the score ranging from 0 (healthy) to 7 (large fibrotic masses). In addition, an ignore class was used to recognize various kinds of non-alveolar tissue, such as fat tissue (example), lymph nodes, large bronchi or blood vessels or air-bubbles in the mounting medium. Scale bar 50 μm.

For the Ashcroft fibrosis model ~14000 512x512 pixel sized images were manually labeled by moving them into folders 0, 1, 3, 5, 7, and ignore (see Fig 2). The folder names correspond to the Ashcroft fibrosis scores; the ignore folder contained non-alveolar tissue with the goal to ignore such regions. For the inflammation model ~3500 images were manually labelled by moving them into folders 0, 1, 2, 3, and ignore. The folder names correspond to the degree of inflammation, defined by the number of inflammatory cells in a field of view. Class 0 corresponds to 0–5 inflammatory cells, class 1 to 6–10, class 2 to 11–20 and class 3 to >21 inflammatory cells. The ignore folder contained regions defined as described above. Before training of both models 90% of the data was randomly selected for training and 10% for validation. Training data are available on an Open Science Framework repository (https://osf.io/28qbc/; DOI 10.17605/OSF.IO/28QBC).

## Results and discussion

Fig 1 shows the general workflow of our deep learning based pathologist’s score approach. The sources were images of lung tissue stained with the Masson´s trichrome stain. This stain is well suited to highlight fibrosis, since it stains connective tissue (blue) differently than other structures (red and purple). To make the large whole slide scans (acquired with 20x magnification) applicable to the deep learning approach, we divided the image into smaller tiles which were inputted to the neural network. We used manually labelled images to train two deep learning models. The first model was trained to recognize the fibrosis Ashcroft score and the second to quantify the degree of inflammation. We decided to train both models on the same type of stain (Masson´s trichrome). This approach allows the recognition of two parameters simultaneously; fibrosis and inflammation, by using one commonly used stain. After training, the CNNs were applicable for classification, yielding spatially resolved maps of fibrosis and inflammation.

In Fig 2 representative images used to train the Ashcroft fibrosis model are shown. In particular the morphological differences in the lower fibrotic range from Ashcroft 0 to Ashcroft 3 are relatively subtle. Moreover, even in the same class the variability of the images is very large. The “ignore” class contains any kind of non-alveolar tissue (e.g., fat, lymph node, large blood vessels or bronchi). Since the network predicts probabilities for the individual Ashcroft scores (see Fig 1), the resulting Ashcroft score will be interpolated between scores in case the neural network is undecided. For example, if Ashcroft scores 1 and 3 would get a probability of 0.5 each, the resulting Ashcroft score would be 2 (0.5*1 + 3*0.5), which is the identical behavior as used in the publication by Ashcroft. [7]

**Fig 3:**
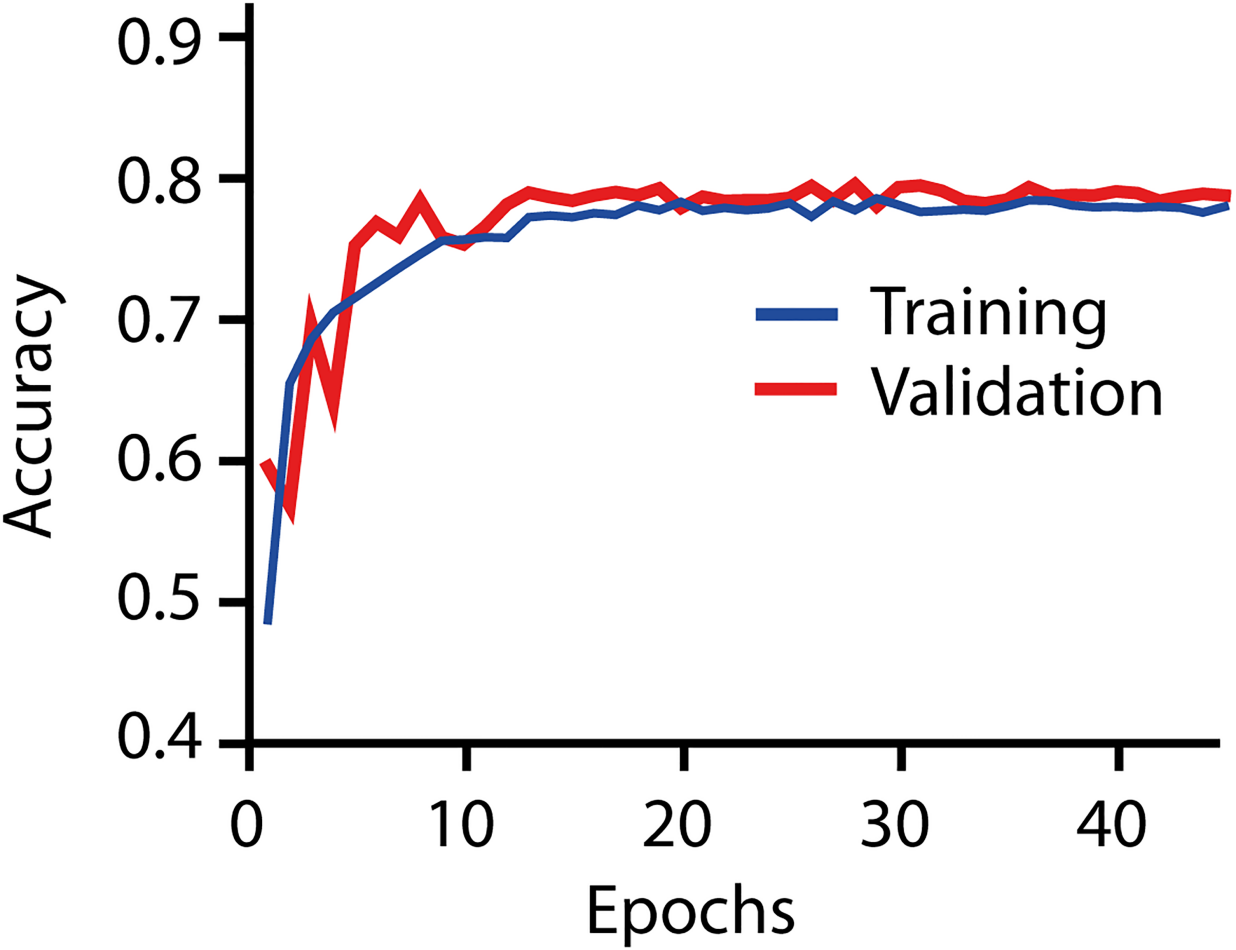
Learning curve of the Ashcroft fibrosis-CNN. The learning curve shows the accuracy of the CNN on the training and validation data vs. the epochs (iterations over the training set). Both curves overlap indicating a good generalization of the CNN to the unseen validation data (i.e. no overfitting).

Using these labeled examples, the convolutional neural network can be trained. Fig 3 shows the learning curve for the Ashcroft fibrosis model. The accuracy of the classifications (percentage of correct classifications) is plotted against the number of times the whole set of training images were shown to the algorithm (epochs). Initially the classification accuracy is low, but the value rapidly increases until it levels off after 10–15 epochs. The final accuracy of the network is A = 79.5% on the validation dataset which was not used for training.

**Fig 4:**
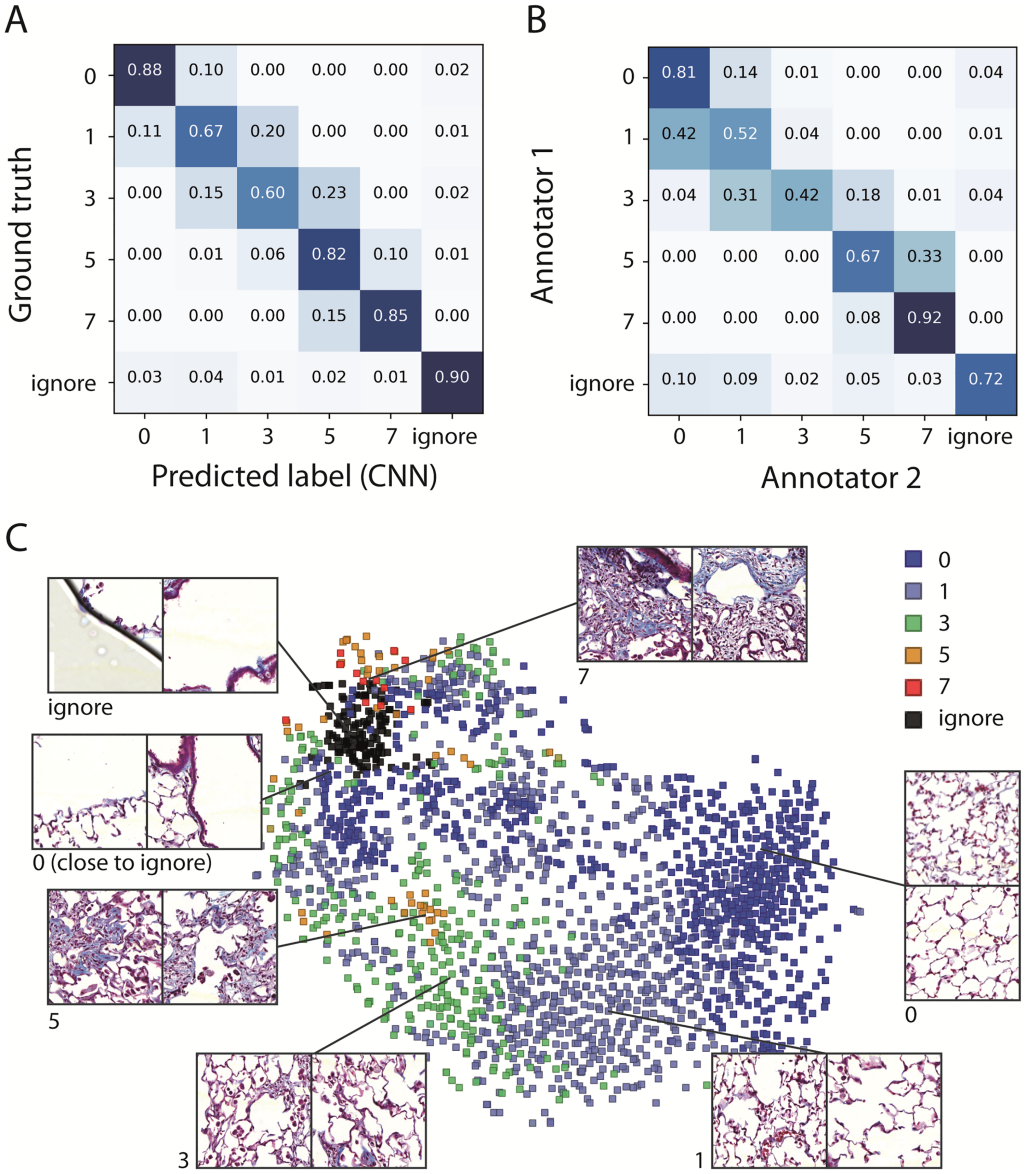
Analyzing annotator agreement and inherent partial ambiguity of image data. **A**. Confusion matrix of predicted labels of the validation data (columns) compared to the ground truth (rows). The numbers are classification probabilities, normalized to a row sum of one. Note that the highest values are either on the diagonal (agreement of ground truth and prediction) or in an element next to the diagonal (a deviation with a neighboring class). The ignore class can be to some extent misinterpreted as all other classes and vice versa. The overall accuracy was A = 79.5%. **B**. Confusion matrix of the agreement of two human experts (annotators 1 and 2) using 400 randomly selected image tiles. The overall result was similar, however the inter annotator agreement of the human experts in terms of accuracy was A = 64.5% and lower than the agreement of the CNN on the unseen validation data. However, the exact value of the agreement of two human experts will depend on the type and amount of training. **C**. Visualization of the inner representation of the image data in the CNN. Here, the last hidden CNN layer representation of the image data was projected in two dimensions with t-SNE, a method to visualize high-dimensional data. Each dot represents one tile from ~2000 validation images. Insets show example images, along with the predicted label and their approximate locations in the cluster. Most classes are separated, however they are interconnected and especially in the transition areas there is ambiguity. Note the smaller area of class 0 tiles close to the “ignore” class (left) showing already properties of the “ignore” class (e.g. an only partially covered tile).

Next we wanted to analyze how the individual classes were recognized to verify if certain Ashcroft scores are more challenging than others. Therefore, we computed a confusion matrix (Fig 4A), which compares the classifications of the validation data which was not used for the network training (columns) with the ground truth of the same data (rows). Most entries are located on the diagonal, where the classified value of a tile matches exactly the ground truth. The most difficult class was the class for an Ashcroft score of 3 (according to the ground truth). In this case 60% of these images were also classified as a three. In 15% of the cases the CNN assigned a score of one and in 23% a score of five. The network was never totally off (zero and seven are both at 0%). Also in case of the other Ashcroft score classes, deviations were almost exclusively found in neighboring values. We argue that this result reflects the inherent ambiguity of the data and the Ashcroft scoring procedure. For many images, the “correct” classes are not perfectly distinct, but overlap with neighboring scores.

We did the same type of analysis with 400 randomly selected images annotated by two human experts (Fig 4B). Qualitatively the result was similar than the comparison of CNN predictions vs the validation data. Also in this case the most challenging class was 3 (42% agreement), and most deviations are with the neighboring scores 1 and 5. However, the overall agreement of the two human expert annotators reached A = 64.5% and was therefore lower than the agreement of the CNN with the validation data (A = 79.5%). Importantly, the result of the human annotators will depend on the type of training and the overall agreement will vary. On the other hand our data shows that CNNs are very performant at learning from a given set of training data provided by a certain annotator. Hence, it is very important to provide high quality gold standard for training of a CNN.

In Fig 4C we visualized the transition between the different classes of the Ashcroft score with t-SNE [28], a method to project the inner representation of an image in the CNN to two dimensions. Each point represents one image tile. Different scores are highlighted by colors and are mostly split in different areas, however the morphological changes from one score to the next are gradual and the transition regions represent the most ambiguous values.

**Fig 5:**
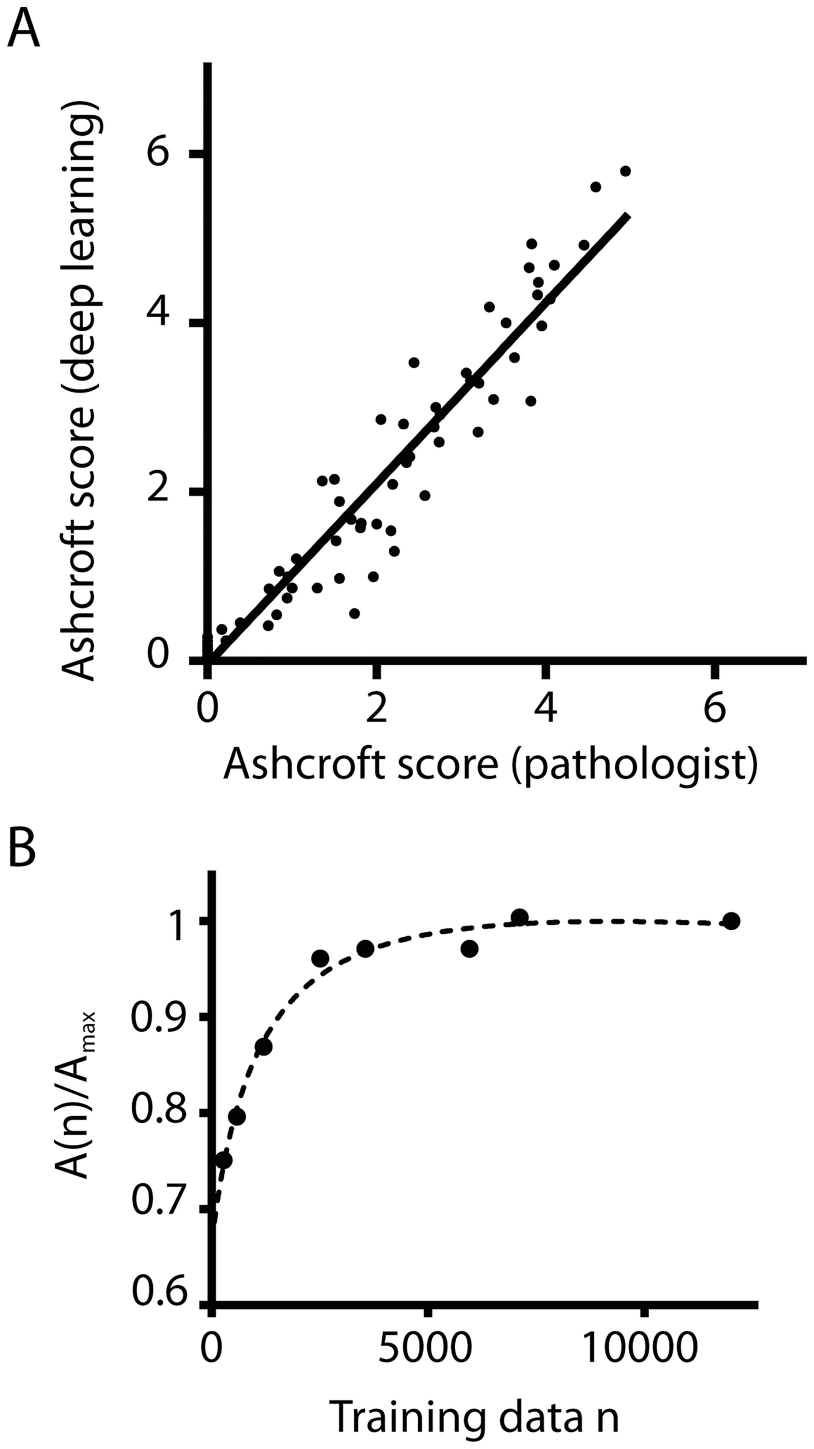
Comparison of CNN and human expert Ashcroft scores and analysis of amount of data required for training. **A**. Comparison of the Ashcroft score performed by a human pathologist vs the Ashcroft scored by the CNN based algorithm. Each value is the mean over a whole lung slice from experiments (*n = 72*) where animals obtained varying doses of Bleomycin to trigger lung fibrosis. Both curves are in good correlation (*r*^*2*^ = 0.92), with a slope m close to 1 and a y-intercept b close to 0 (*m* = 1.07 ± 0.04, *b* = -0.04 ±0.08, fit parameters are optimum fit parameters ± the difference at the 5% and 95% confidence intervals.) **B**. Dependency of the accuracy of the Ashcroft fibrosis CNN model on the amount of training data available. *A(n) / A*_*max*_ compares the accuracy *A(n)* of a model trained using n randomly selected images to the accuracy *A*_*max*_ of a model trained all available labelled images (*n* = 12000). The dashed line is an empirical fit using an asymptotic function.

We then compared the CNN based Ashcroft score to the Ashcroft score of an experienced pathologist (Fig 5A). Both scores were in very good agreement with a squared Pearson’s coefficient r^2^ = 0.92, a slope close to 1 (m = 1.07 ± 0.04) and a y-intercept close to 0 (b = -0.04 ±0.08). This shows that our trained CNN classifies the Ashcroft scores comparable to a trained pathologist. In particular, also the low Ashcroft score regime from 0–3 could be discriminated by our CNN. Previously, this lower Ashcroft value range could not be resolved with classical computer vision approaches (compare for example [8]), presumably due to the very subtle morphological changes in this region (see Fig 2).

Nonetheless, it is crucial to regularly verify the CNN-based results by a human expert. In particular, if novel images are analyzed which may contain morphological features, which were previously not sufficiently represented in the training data. Similarly, milder forms of preparation artifacts such as slight tissue compression or flipping of alveolar walls might affect the scores. In these cases it is recommended to incorporate such types of images in the training data and retrain the network until the classification agrees with the expert.

The generation of training data is time-consuming and can be a limiting factor in the development of novel models. Therefore, we analyzed how many images are required for the training of the Ashcroft fibrosis model. We varied the amount of training images, and analyzed the maximal classification accuracy (corresponding to the plateau accuracy in Fig 3) on an unseen validation data set (Fig 5B). Initially, the relative accuracy increases quickly with the amount of training data. However, over a certain threshold, adding more data does not markedly improve the final accuracy.

The exact shape of the data vs accuracy relation will depend on the nature of the data (e.g. how many classes are used and how difficult to separate the image classes are) and the details of the CNN setting (e.g. how much augmentation is used and the type of CNN). Therefore, this shape should be determined when training a new type of classifier. This allows optimization of the procedure, using an adequate amount of data, but minimizing the time required to generate training data.

**Fig 6:**
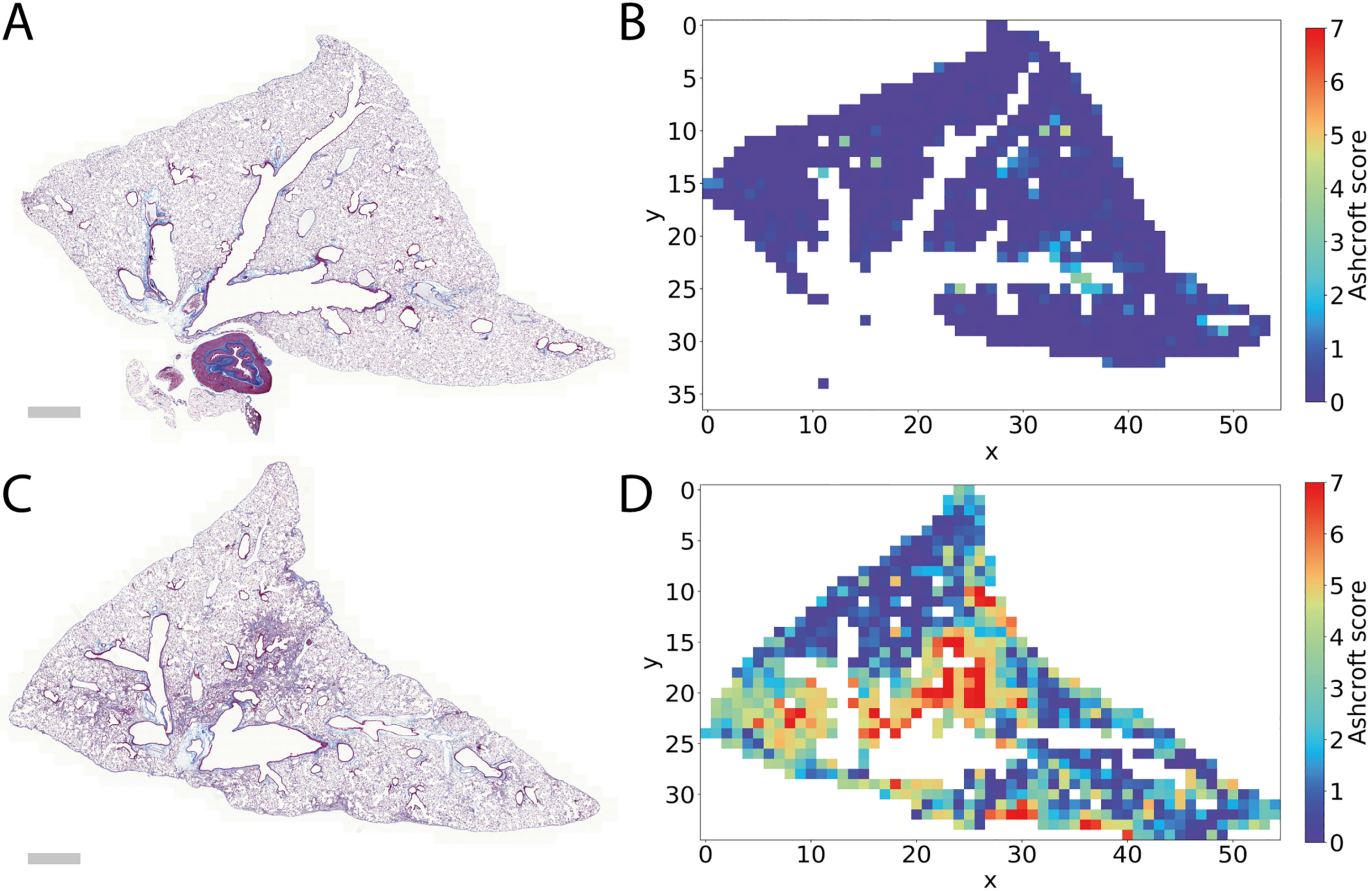
Whole slide scan of Masson´s trichrome stained mouse lungs and corresponding color coded Ashcroft fibrosis scores. **A** and **B** represent a lung sample with no fibrosis. Note that the lymph node (dense structure at bottom center of **A**) is not considered in the Ashcroft score image, since it is recognized as to be ignored. **C** and **D** represent an image and the corresponding Ashcroft map of a fibrotic lung. Scale bar 1 mm.

The trained Ashcroft fibrosis CNN can generate maps of the Ashcroft fibrosis score, as shown in Fig 6. Such visualizations could support further insights into the mode of action or application of tested compounds. For example, improvements in fibrotic scores might be located next to blood vessels or next to large bronchi where the first entry points of compounds could be situated.

We also wanted to add a further layer of information and thus built a second model: the CNN inflammation model. The goal was to quantify inflammatory processes by using the same type of images stained with Masson´s trichrome. The advantage of this approach is that both scores would be available by using a single type of image stained with a relatively simple stain without the need to analyze consecutive slides with an immunohistochemistry staining of immune cells (e.g. by labelling a pan leukocyte marker such as CD45 [30]).

**Fig 7:**
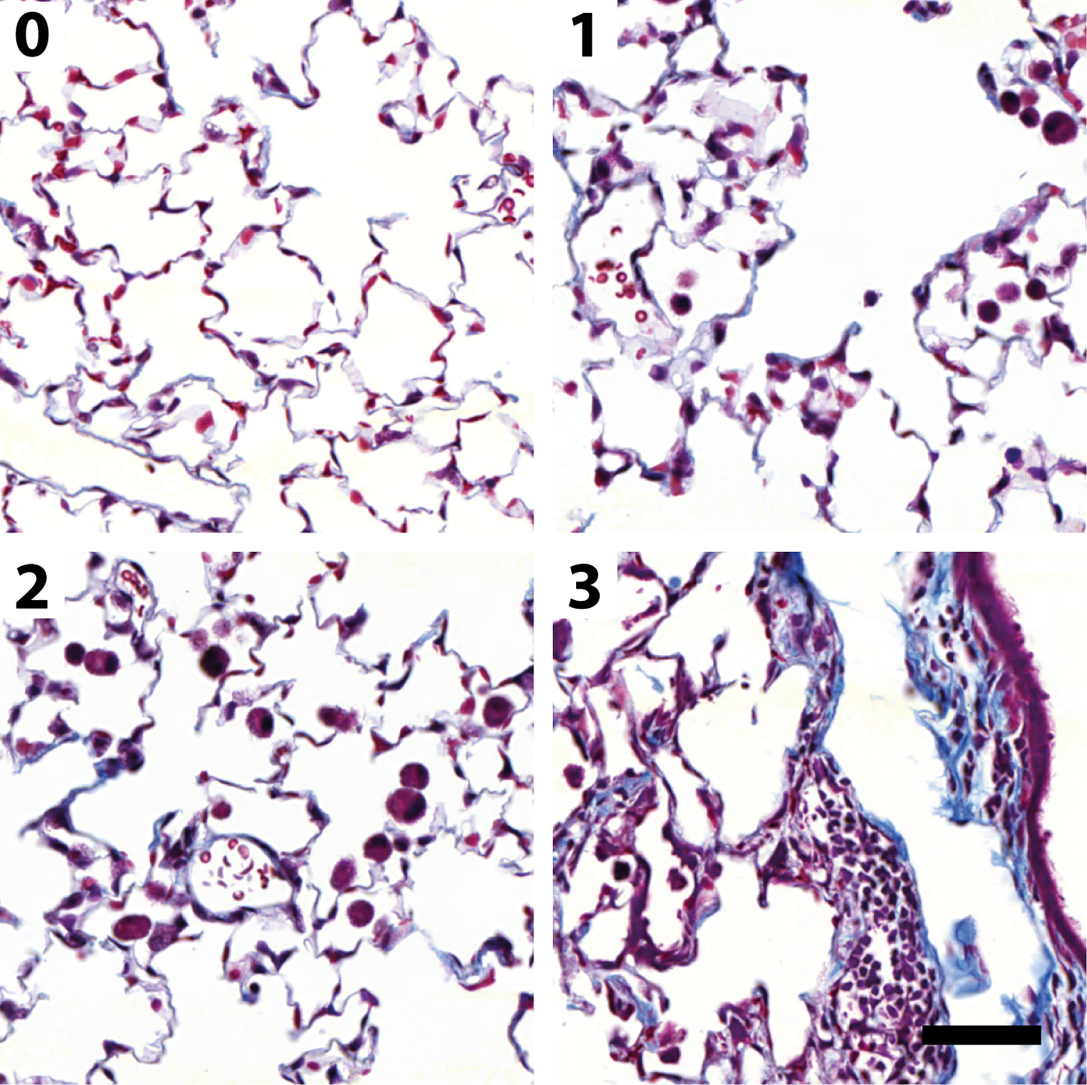
Representative tiles to illustrate the inflammation score. Numbers represent the inflammation score. The score is defined by the number of inflammatory cells in a field of view (0: 0–5, 1: 6–10, 2: 11–20, 3: above 20 inflammatory cells). In addition a ignore class was defined, as shown in Fig 2 (not shown). Scale bar 50 μm.

We defined our novel inflammation score ranging from 0–3 by using the number of inflammatory cells in a field of view, irrespective of the type of cell. The cell densities corresponding to scores of 0–3 were empirically selected such that they cover the range of typical experimentally observed situations from non-inflamed (score 0) to severe inflammation (score 3) and provide the possibility to distinguish intermediate inflammation degrees (scores 1 and 2). Example images for the training classes used for the inflammation model are shown in Fig 7. Particular attention was given to the composition of the training data, such that it reflected a large variety of inflammatory cells in the respective inflammatory classes. Further, images containing a variety of non-inflammatory cell types such as erythrocytes or pneumocytes were incorporated in all classes of the training data.

**Fig 8:**
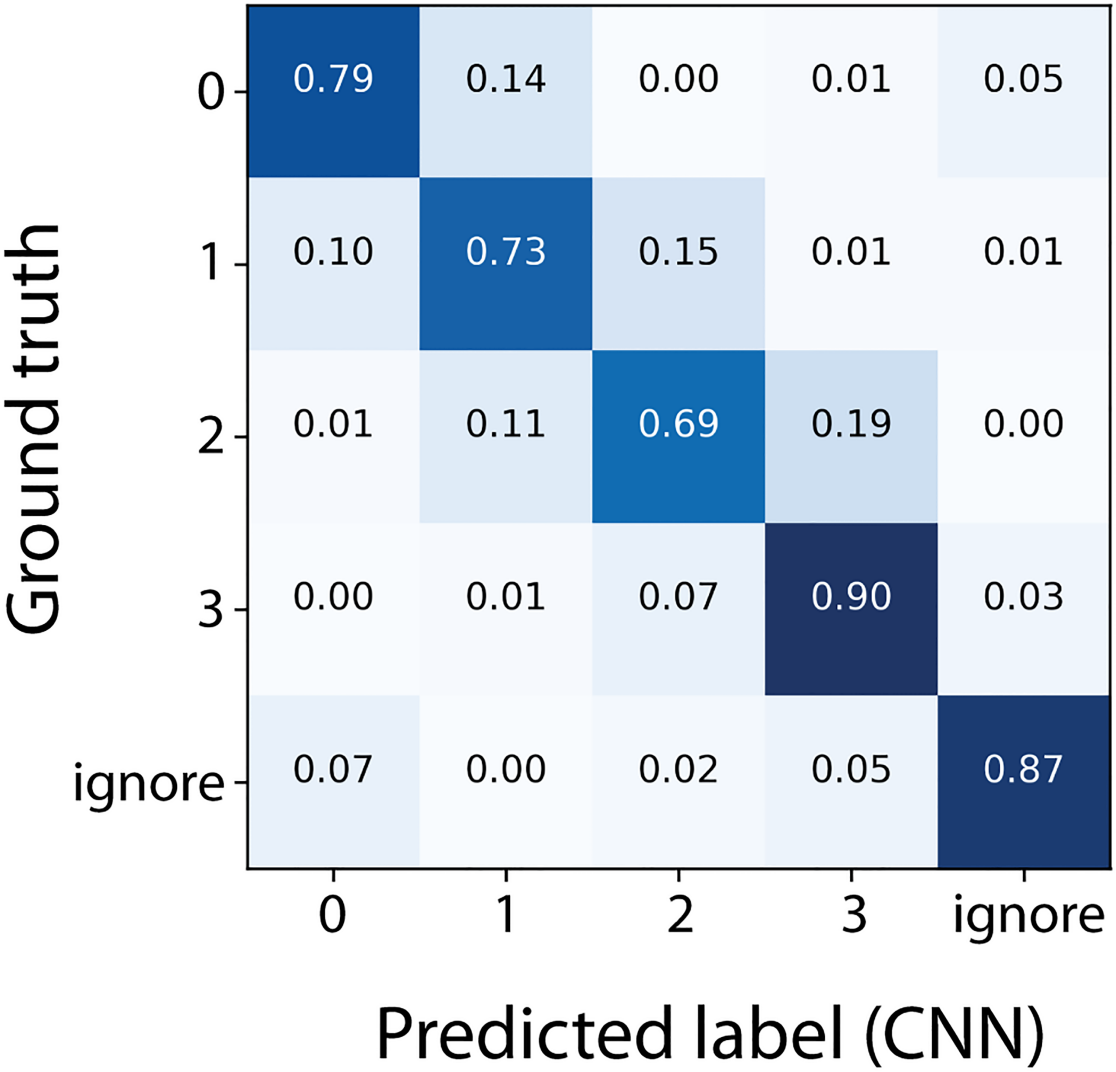
Confusion matrix comparing the classifications of the inflammation score CNN vs the ground truth. Classifications of the validation data (columns) are compared to the ground truth (rows). Numbers are classification probabilities, normalized to a row sum of one. The highest values are mostly on the diagonal (agreement of ground truth and prediction) or in an element next to the diagonal (a deviation with a neighboring class).

**Fig 9:**
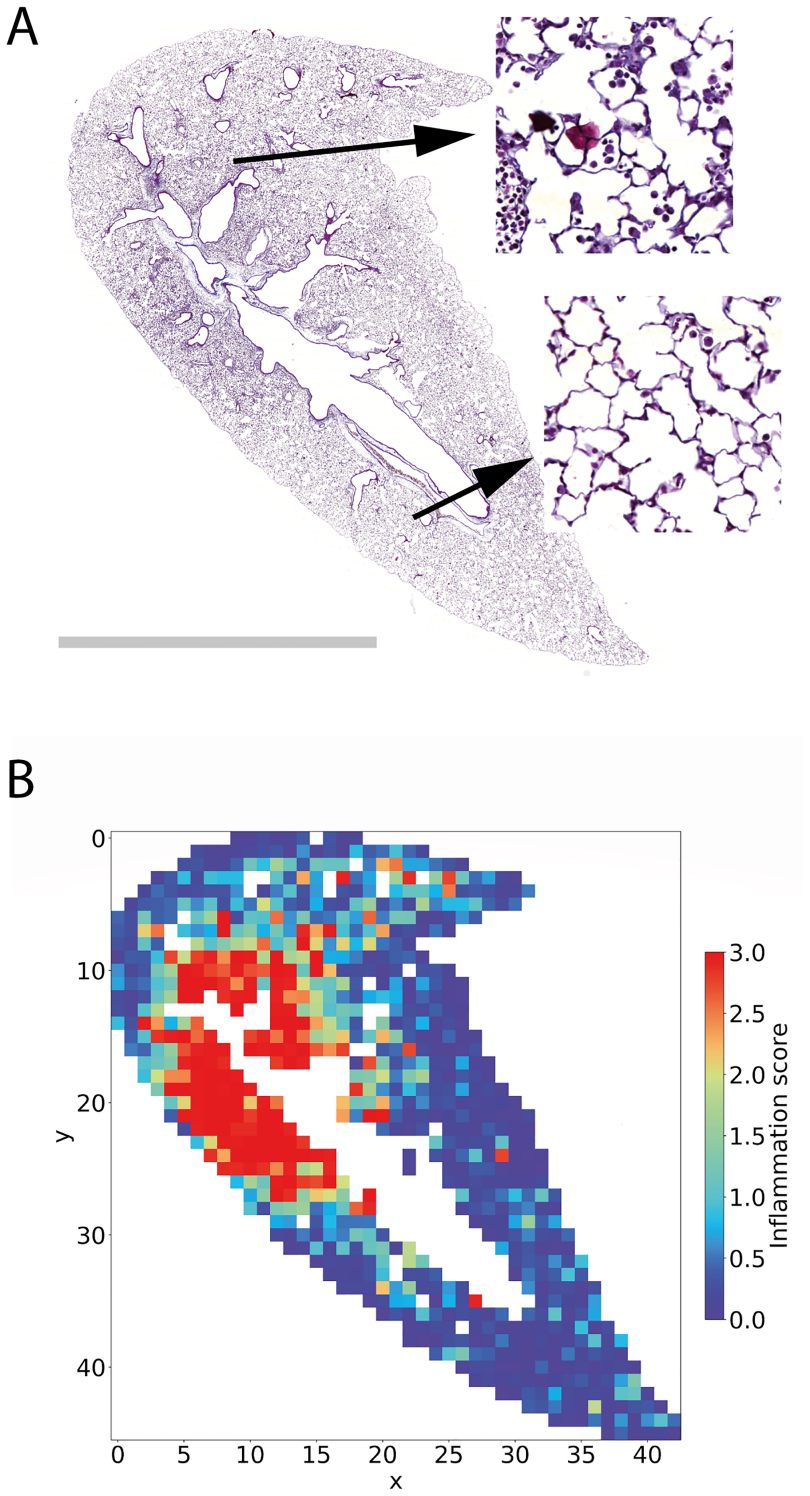
Example of the spatial inflammation score. **A**. Whole slide scan of a mouse lung with inserts showing a more inflamed tile (top) and a non-inflamed tile (bottom). Scale bar 5 mm. **B**. Corresponding color coded map of the inflammation score from 0–3.

After training, the final accuracy of the lung inflammation CNN was A = 80.0%. To characterize the model further, the confusion matrix was analyzed (Fig 8). Most classifications agree with the ground truth (values situated on the diagonal). Deviations are almost exclusively found in neighboring classes, reflecting again a partial ambiguity of the class assignments of these images, e.g. in cases where it is hard to decide if certain cells are inflammatory or not or in cases where parts of the cells are on the edge of the image. The most difficult class 2 was correctly classified with 69% accuracy, which was higher than the accuracy for the most difficult class 3 in the Ashcroft fibrosis model (A = 60%). Presumably, this is related to a lower degree of inherent ambiguity of the inflammation classes compared to the Ashcroft fibrosis model. Moreover, the identification of immune cells may be an easier task for the CNN as compared to fibrosis classification (i.e. identifying and counting cells is a simpler task than assessing complex fibrotic changes). Fig 9A shows an exemplary image of a lung and the corresponding map of the inflammation score in Fig 9B. In this sample both, healthy and a larger inflammatory region are visible. The insets in Fig 9A show two exemplary cases of an inflamed and a not inflamed spot.

## Conclusion

The recent advances in object recognition by using CNNs allow that these algorithms now perform tasks which were previously the exclusive domain of human experts. We showed this in case of the Ashcroft fibrosis score on mouse tissue, where our CNN based score resulted in practically identical values to the pathologist’s scores. Moreover, CNNs can now be used to generate novel scores such as the inflammation score shown here. The inflammation CNN is based on recognizing features on the same type of Masson´s trichrome stained image as used for the Ashcroft fibrosis CNN and no expensive immunohistochemistry stains were required. The inflammation score relies on a large diversity of morphological features which hinders the generation of similar readouts with classical image analysis. Also, manual evaluation is impractical due to the long time needed for analysis.

We assume that in the next years such CNN based scores will become increasingly available in histology. Furthermore, CNNs will also support readouts from in vitro models such as organs on a chip or cell culture systems.

Eventually, such deep learning based scorers will help to automate time consuming tissue scoring tasks. First of all, this will allow the experts to focus more on the complex and creative parts of science. Second, scoring will no longer be a bottleneck task and more samples can be quantitatively analyzed with established scores but also with completely new readouts.
